# Sequence-specific ^1^H, ^13^C and ^15^N backbone NMR assignments for the N-terminal IgV-like domain (D1) and full extracellular region (D1D2) of PD-L1

**DOI:** 10.1007/s12104-022-10092-5

**Published:** 2022-06-08

**Authors:** Kayleigh Walker, Lorna C. Waters, Geoff Kelly, Frederick W. Muskett, Mark D. Carr

**Affiliations:** 1 Leicester Institute of Structural and Chemical Biology, Leicester, UK; 2 Department of Molecular and Cell Biology, University of Leicester, Henry Wellcome Building, Lancaster Road, Leicester, LE1 7HB UK; 3 Medical Research Council (MRC) Biomedical NMR Centre, The Francis Crick Institute, Midland Road, London, NW1 1AT UK

**Keywords:** Programmed cell death-ligand 1, Immune checkpoint, Cancer

## Abstract

The co-inhibitory immune checkpoint interaction between programmed cell death-protein 1 (PD-1) and programmed cell death-ligand 1 (PD-L1) serves to regulate T-cell activation, promoting self-tolerance. Over-expression of PD-L1 is a mechanism through which tumour cells can evade detection by the immune system. Several therapeutic antibodies targeting PD-L1 or PD-1 have been approved for the treatment of a variety of cancers, however, the discovery and development of small-molecule inhibitors of PD-L1 remains a challenge. Here we report comprehensive sequence-specific backbone resonance assignments (^1^H, ^13^C, and ^15^N) obtained for the N-terminal IgV-like domain of PD-L1 (D1) and the full two domain extracellular region (D1D2). These NMR assignments will serve as a useful tool in the discovery of small-molecule therapeutics targeting PD-L1 and in the characterisation of functional interactions with other protein partners, such as CD80.

## Introduction

Human PD-L1 is a 272 amino acid, single-pass transmembrane protein, with two N-terminal Ig-like domains forming the extracellular region (residues 19–239). PD-L1 is the ligand for PD-1 (265 residues), which is also a single-pass transmembrane protein, with a single Ig-like domain forming the extracellular region (amino acids 24–170). The interaction between PD-1 and PD-L1 has been shown to be facilitated by the most N-terminal IgV-like domain of PD-L1 (D1) and the extracellular Ig-like domain of PD-1 (Lin et al. 2008), which regulates a key immune checkpoint, promoting self-tolerance and protecting from auto-immune responses. These effects are achieved by modulating the threshold of T-cell activation via intracellular signalling through PD-1 expressed on T-cells, which is inhibitory to T-cell Receptor signalling (Okazaki and Honjo 2007).

Over-expression of human PD-L1 by tumour cells has been shown to be a key mechanism by which cancers can evade detection by the immune system (Freeman et al. 2000) and has been seen on the surface of many different tumour types, including melanoma, non-small cell lung cancer and lymphoma (Konishi et al. 2004; Nakanishi et al. 2007). Targeting immune checkpoint regulators such as PD-1 and PD-L1 with monoclonal antibodies has revolutionised the treatment of a number of cancers (Topalian et al. 2012; Brahmer et al. 2012; Robert et al. 2015). To date, seven antibodies selected for potent inhibition of PD-1 binding to PD-L1 have been approved as therapeutics, with three targeting the extracellular region of PD-L1 (Upadhaya et al. 2022). Despite highly beneficial clinical responses there are several drawbacks associated with the therapeutic antibodies, including adverse auto-immune effects due to long half-lives in vivo (Naidoo et al. 2015) and problems with tumour penetration (Tan et al. 2016). The discovery and development of specific small-molecule inhibitors of the interaction of PD-L1 with PD-1 has the potential to overcome these problems but remains a major challenge.

PD-L1 has also been found to bind to the extracellular region of membrane-bound CD80 (residues 35–242), which appears to be limited in vivo to when both proteins are on the surface of the same cell (Chaudri et al. 2018). The interaction of PD-L1 with CD80 has been shown to prevent PD-L1 binding to PD-1 and is therefore stimulatory to T-cell responses (Sigiura et al. 2019). The comprehensive backbone NMR assignments reported for the extracellular region of PD-L1 here are expected to be useful tools for both small molecule drug discovery and for further characterisation of interactions with functional partner proteins such as CD80.

## Methods and experiments

### Protein expression and purification

The coding regions for human PD-L1 (D1: 19–134) and PD-L1 (D1D2: 19–239) were synthesised and cloned into pET28a by GenScript, with codon usage optimised for expression in *Escherichia coli.* For triple resonance NMR experiments, uniformly ^15^N/^13^C-labelled PD-L1 (D1) and ^2^H/^15^N/^13^C-labelled PD-L1 (D1D2) were expressed as insoluble inclusion bodies in appropriately transformed *E* *coli *BL21 (DE3) cells grown in M9 minimal medium containing 3 g/l ^13^C glucose and 1 g/l ^15^N ammonium sulphate. Deuterated samples were prepared from cells grown in minimal media prepared using > 99% D_2_O. The BL21 (DE3) cells were cultured at 37° C and protein expression was induced with 1 mM IPTG at an optical density of 0.8–1.0 at 600 nm. The cells were then cultured for a further 5 hours before harvesting by centrifugation. For refolding and purification of PD-L1, cell pellets were resuspended in PBS at pH 7.2 and lysed by sonication before the inclusion bodies were collected by centrifugation. Inclusion bodies were washed twice with 50 mM tris-HCl, 200 mM NaCl, 0.5% triton-X100, 10 mM EDTA and 10 mM DTT at pH 8.0 and once in the same buffer without triton-X100. Washed inclusion bodies were then resolubilised in 50 mM tris-HCl, 5 M guanidine-HCl, 200 mM NaCl and 20 mM DTT at pH 8.0 prior to refolding by drop-wise 100-fold dilution into 0.1 M tris-HCl, 1 M arginine, 0.25 mM oxidised glutathione, and 0.25 mM reduced glutathione at pH 8.0 for PD-L1 (D1) (Zak et al. 2015) and for PD-L1 (D1D2) into the same buffer but with 0.5 mM oxidised glutathione and 2 mM reduced glutathione. After slow stirring for 18 hours at 4° C, refolding mixtures were concentrated by tangential flow filtration, dialysed into a 25 mM potassium phosphate, 20 mM sodium chloride, 10 μM EDTA and 0.02% sodium azide (w/v) buffer at pH 7.5 prior to final purification by size exclusion chromatography on a Superdex-75 column (GE Healthcare).

### NMR spectroscopy

3D NMR experiments were acquired from 190 μM and 240 μM samples of uniformly ^15^N/^13^C labelled PD-L1 (D1) and ^2^H/^15^N/^13^C labelled PD-L1 (D1D2) respectively, in a 25 mM potassium phosphate, 20 mM sodium chloride, 10 μM EDTA and 0.02% sodium azide (w/v) buffer at pH 7.5, containing 5% D_2_O. All NMR experiments for PD-L1 (D1) were collected at 298 K whereas NMR experiments for PD-L1 (D1D2) were recorded at 303 K. Sequential backbone resonance assignments for PD-L1 (D1) were determined using a combination of ^15^N/^13^C/^1^H HNCACB (Wittekind and Mueller 1993), CBCA(CO)NH (Grezesiek and Bax 1992) and HNCO (Kay et al. 1990) spectra. Assignments for PD-L1 (D1D2) were determined using a combination of ^15^N/^13^C/^1^H TROSY-HNCACB, TROSY-HN(CO)CACB (Yamazaki et al. 1994) and TROSY-HNCO spectra. Linear prediction was used to extend the effective acquisition time in ^15^N by two fold in the CBCA(CO)NH and HNCO experiments for PD-L1 (D1) and in the TROSY-HNCO for PD-L1 (D1D2). The acquisition parameters used for all NMR experiments are summarised in Table 1. NMR experiments were processed using NMRPipe (Delaglio et al. 1995). Where non-uniform sampling (NUS) was used during data collection, the data was reconstructed using NMRPipe’s Interative Shrinkage Thresholding (IST) software. All analysis of spectra was carried out manually using NMRFAM-SPARKY (Lee et al. 2015).

**Table 1:** A summary of the acquisition parameters used in the NMR experiments recorded to obtain backbone NMR assignments for PD-L1 (D1) and PD-L1 (D1D2)

	AQ (ms)			SW (ppm)			MHz	NS	NUS (%**)**
	H	N	C	H	N	C			
PDL1 (D1)									
HSQC	100	50	–	12	32	–	800	8	–
HNCO	62	18	25	12	32	14	800	8	–
HNCACB	77	25	9	14	32	75	950	16	26
CBCA(CO)NH	80	22	6	12	32	75	800	8	–
PDL1 (D1D2**)**									
TROSY-HSQC	134	93	–	12	28.5	–	950	32	–
TROSY-HNCO	100	22	20	12	28.6	14	800	16	–
TROSY-HNCACB	90	22	6	12	28.5	80	950	24	25
TROSY-HN(CO)CACB	67	22	3	12	28.5	80	950	48	25

### Extent of assignments and data deposition

**Fig. 1:**
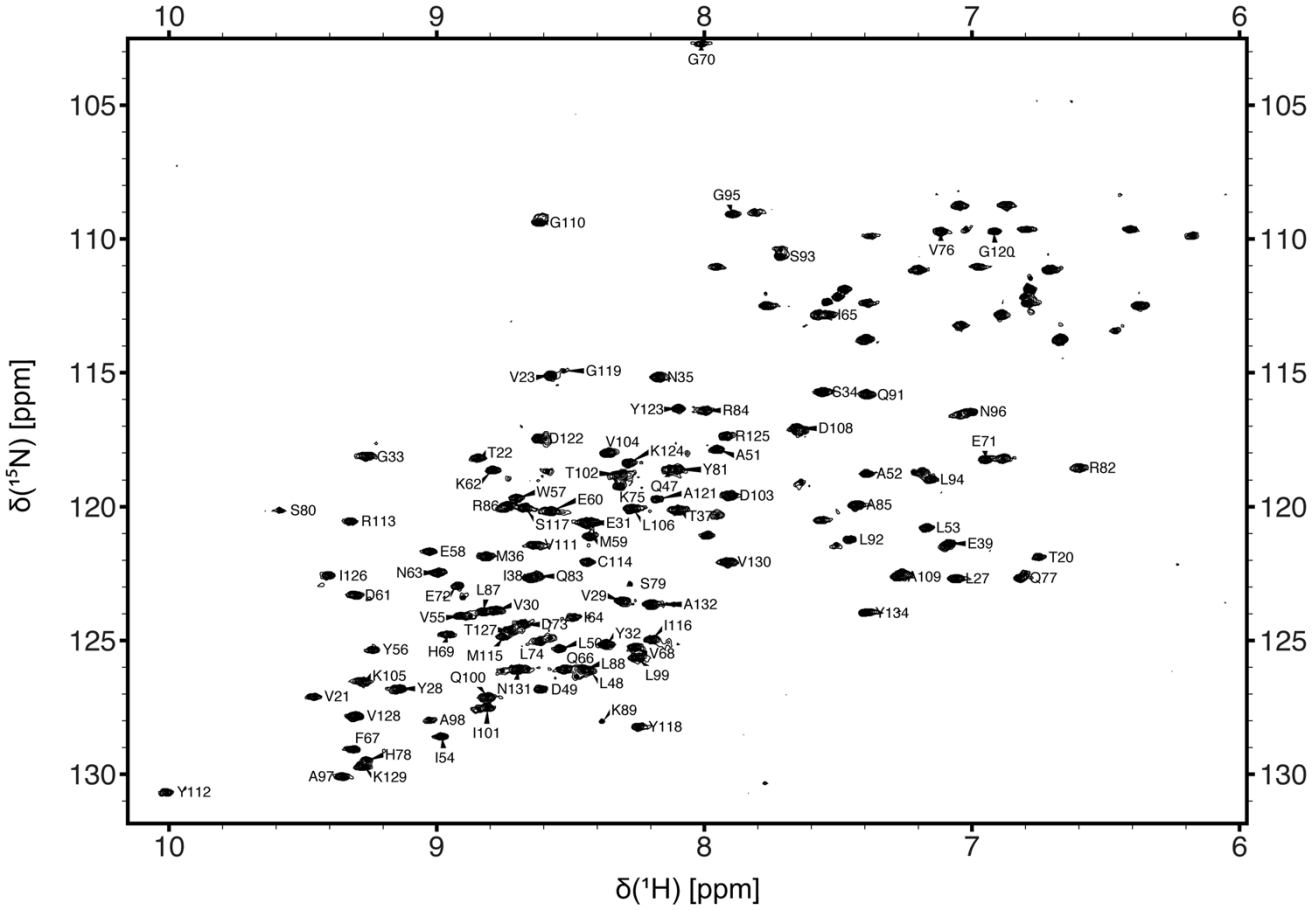
A typical ^15^N/^1^H HSQC spectrum of PD-L1 (D1) (190 µM) in a 25 mM potassium phosphate, 20 mM sodium chloride, 10 μM EDTA and 0.02% sodium azide (w/v) buffer at pH 7.5 containing 5% D_2_O. The spectrum was acquired at 298 K and 800 MHz. The sequence-specific assignments obtained for backbone amide signals are indicated in the contour plot

**Fig. 2:**
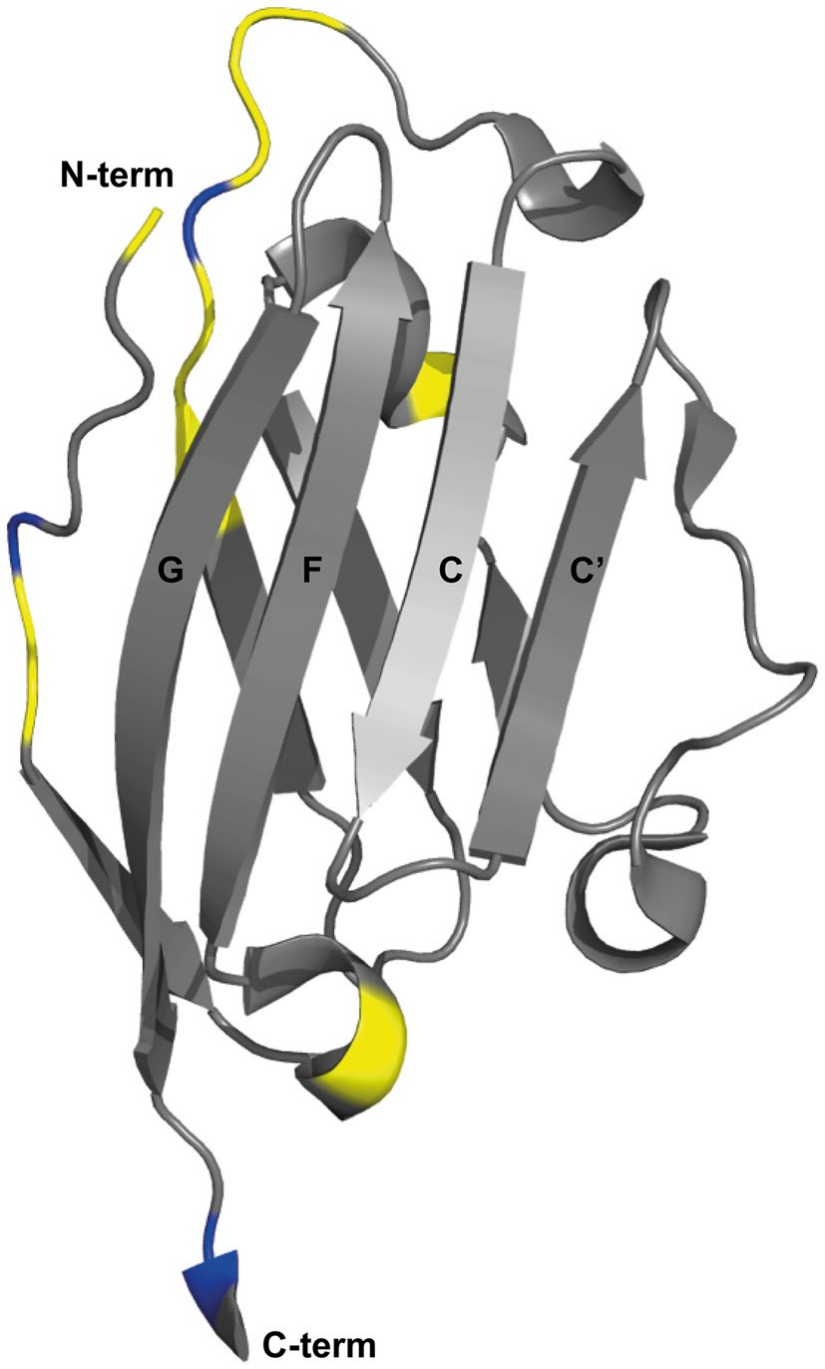
A ribbon representation of the backbone topology of PD-L1 (D1) (PDB 5C3T), with the positions of residues with non-assigned backbone amide groups highlighted in yellow. The locations of proline residues are shown in blue

Comprehensive sequence-specific backbone resonance assignments (90% H^N^, 90% N, 90% C^’^, 92% Cα and 92% Cβ) were obtained for PD-L1 (D1) with backbone amide assignments made for 101 of the 112 non-proline residues (Fig. 1). For the majority of the residues in PD-L1 (D1) with unassigned backbone amide signals this is due to associated peaks being absent from the NMR spectra acquired due to conformational dynamics, resulting in substantial broadening of amide resonances. For example, backbone amide signals could not be assigned for residues C40 to K46, which form part of a long solvent accessible loop connecting β-strands B and C in PD-L1 (D1) (Fig. 2) with the potential for significant conformational heterogeneity. The sequence-specific backbone NMR assignments obtained for PD-L1 (D1) have been deposited in the BioMagResBank (http://www.bmrb.wisc.edu) under accession number 51412.

**Fig. 3:**
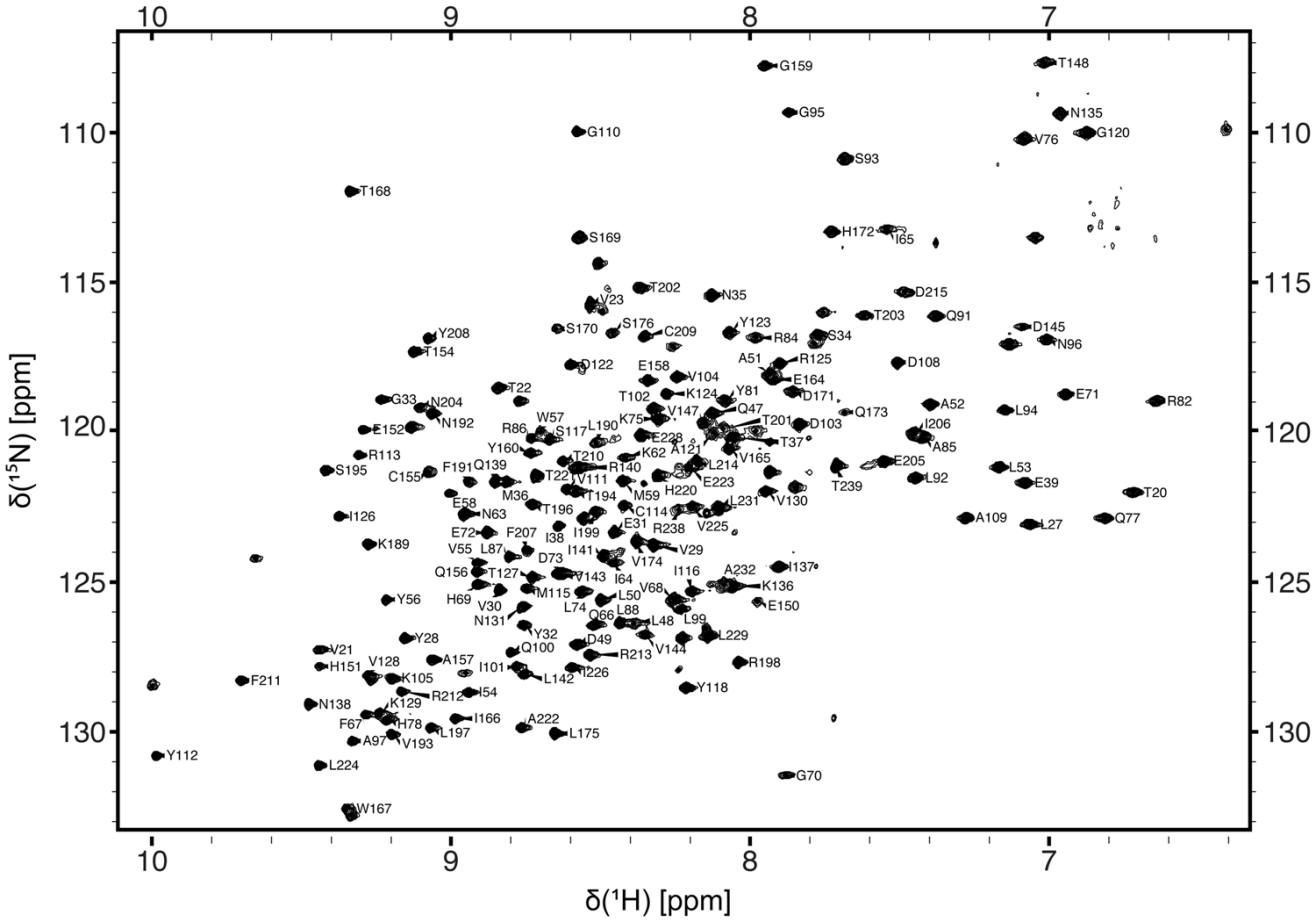
A typical ^15^N/^1^H TROSY spectrum of PD-L1 (D1D2) (240 µM) in a 25 mM potassium phosphate, 20 mM sodium chloride, 10 μM EDTA and 0.02% sodium azide (w/v) buffer at pH 7.5 containing 5% D_2_O. The spectrum was acquired at 303 K and 950 MHz. The sequence-specific assignments obtained for backbone amide signals are indicated in the contour plot

**Fig. 4:**
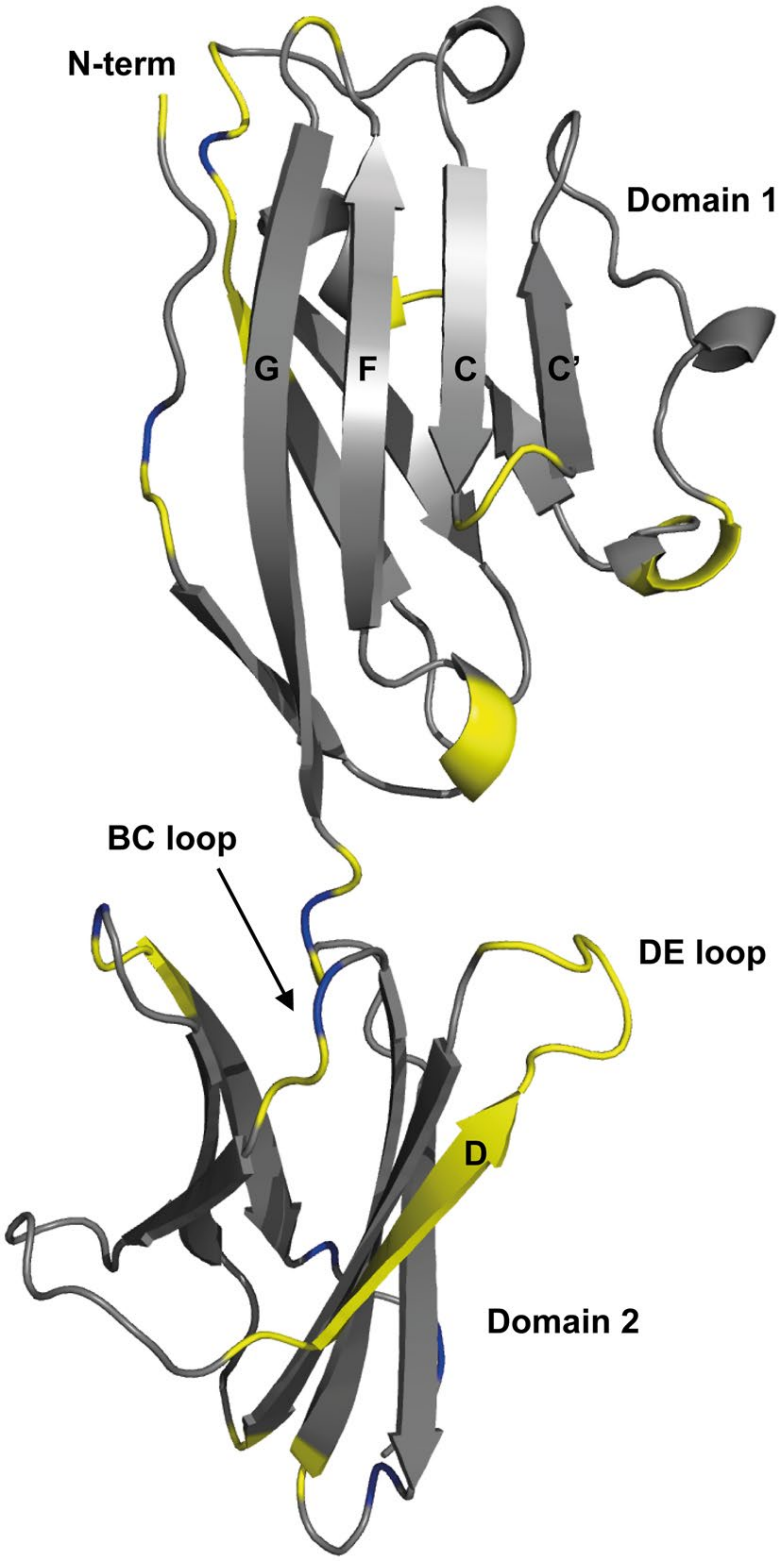
A ribbon representation of the backbone topology of PD-L1 (D1D2) (PDB: 3FN3), with the positions of residues with non-assigned backbone amide groups highlighted in yellow. The locations of proline residues are shown in blue

Somewhat less complete sequence specific backbone assignments were obtained for PD-L1 (D1D2) (79% H^N^, 79% N, 75% C^’^, 80% Cα and 79% Cβ), with backbone amide assignments made for 167 of the 211 non-proline residues (Fig. 3). This is primarily due to assignment of only 75% of the backbone amide resonances expected from the membrane proximal domain of PD-L1 (D2). The non-assigned signals are predominantly associated with residues forming the loops connecting β-strands B and C, D and E, and β-strand D (Fig. 4). Given the absence of these signals in the 3D NMR spectra, it seems likely that this region of PD-L1 (D2) is exchanging between multiple conformational states and/or exchanging with the solvent, resulting in broadening of NMR signals beyond detection. The sequence-specific backbone NMR assignments obtained for PD-L1 (D1D2) have been deposited in the BioMagResBank under accession number 51411.

**Fig. 5:**
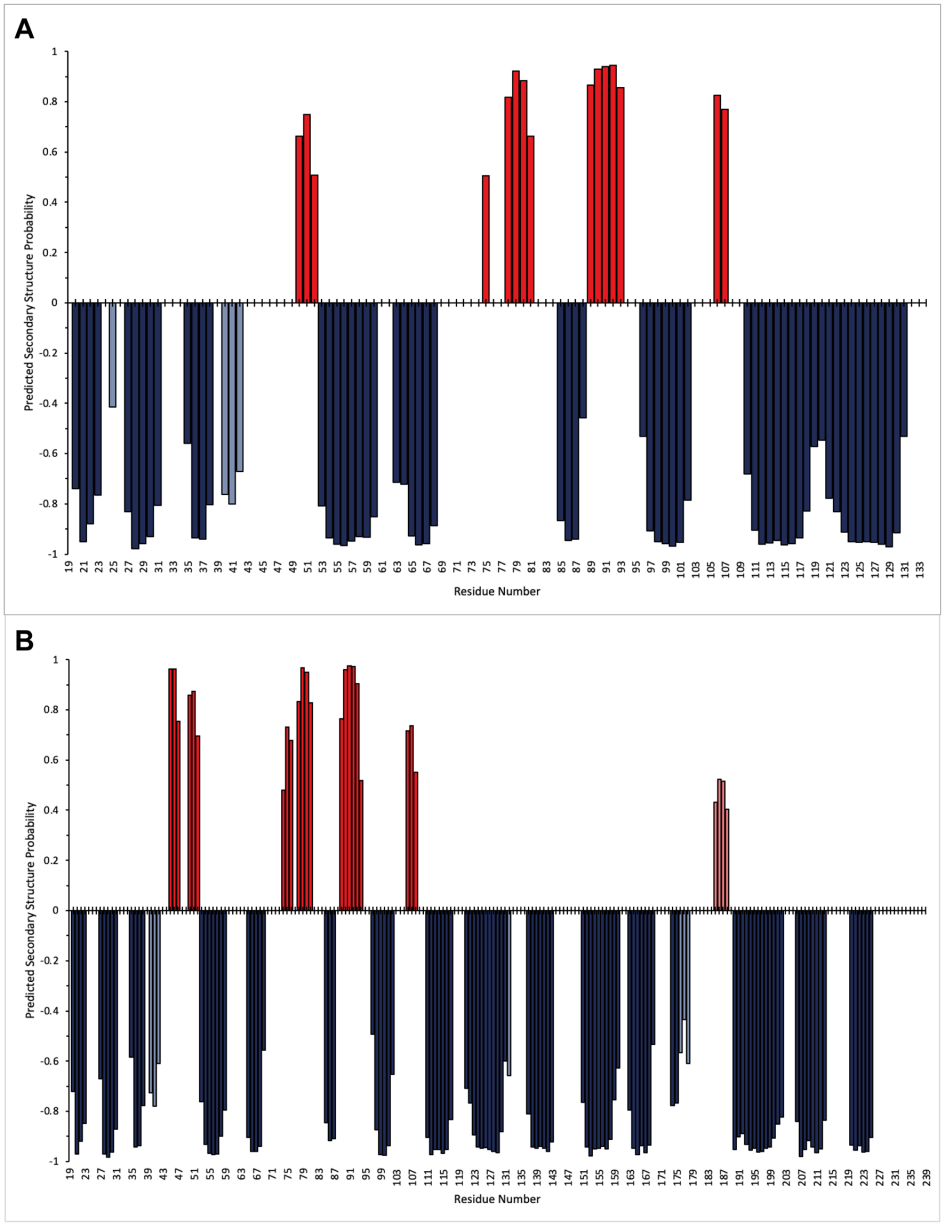
Predicted secondary structure of PD-L1_D1 (A) and PD-L1_D1D2 (B) based on the analysis of sequence-specific backbone NMR assignments (N, NH, CO, Cα, and Cβ) obtained for both proteins using TALOS-N. Predicted ⍺-helical structure is shown as positive values (red) and predicted β-strands as negative values (blue). For a limited number of residues, the predicted secondary structure is based on the protein sequence alone, which is indicated by paler red or blue bars

**Fig. 6:**
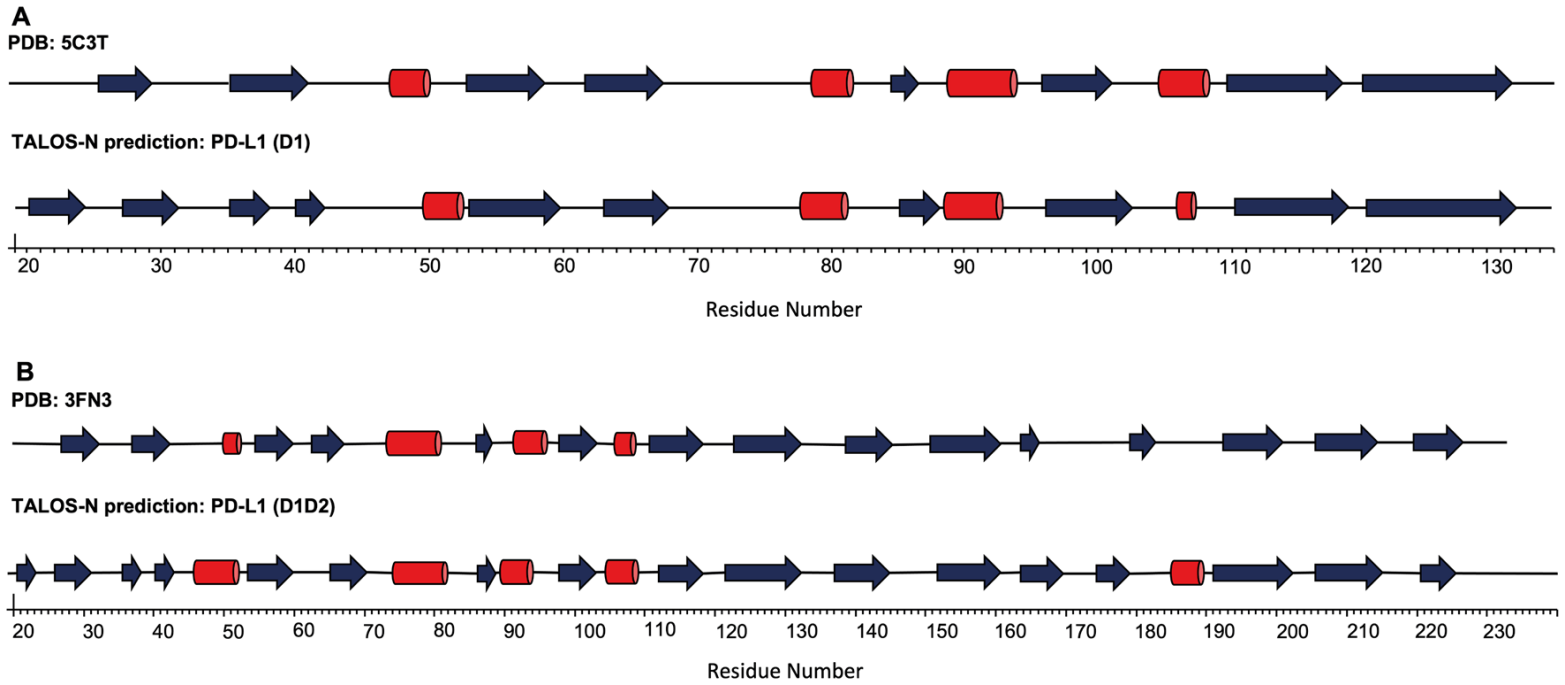
Comparison of TALOS_N secondary structure prediction and reported crystallographic structures of PD-L1 (D1) (**A**) and PD-L1 (D1D2) (**B**). ⍺-helical structure is shown as red cylinders and β-strand structure is shown as blue arrows

TALOS-N was used to predict the secondary structure of both PD-L1 (D1) and PD-L1 (D1D2) using primarily the NMR assignments obtained (Shen and Bax 2013), however, predictions for non-assigned residues were based on the protein sequence (Fig. 5). For PD-L1 (D1D2), the NMR-based predictions show good agreement with the crystal structure reported for PD-L1 (D1D2) (Fig. 6), PDB: 3FN3, (Chen et al. 2010). TALOS-N analysis of the NMR data predicts an additional α-helix from K185-E188, however, for these residues this is based on sequence alone. In the case of PD-L1 (D1), the secondary structure predicted by analysis of the NMR data also showed good agreement with the crystal structure reported for PD-L1 (D1) (Fig. 6) PDB: 5C3T (Zak et al. 2015).

It is our hope that these backbone NMR assignments will be a useful tool for the study of the interactions of PD-L1 with functional partners as well as in the development of small-molecule therapeutics targeting PD-L1.

## Data Availability

The sequence-specific backbone NMR assignments obtained for PD-L1 (D1) have been deposited in the BioMagResBank (http://www.bmrb.wisc.edu) under accession number 51412. The sequence-specific backbone NMR assignments obtained for PD-L1 (D1D2) have been deposited in the BioMagResBank under accession number 51411.
